# Pathogen recognition by NK cells amplifies the pro-inflammatory cytokine production of monocyte-derived DC via IFN-γ

**DOI:** 10.1186/s12865-018-0247-y

**Published:** 2018-02-13

**Authors:** Tammy Oth, Thomas H. P. M. Habets, Wilfred T. V. Germeraad, Marijke I. Zonneveld, Gerard M. J. Bos, Joris Vanderlocht

**Affiliations:** 1 Division of Hematology, Department of Internal Medicine, School of Oncology and Developmental Biology, Maastricht University Medical Center+, Maastricht, the Netherlands; 2 Central Diagnostic Laboratory, Division of Immunology, Maastricht University Medical Center+, Maastricht, the Netherlands; 3 MAASTRO Laboratory, Department of Radiation Oncology, School of Oncology and Developmental Biology, Maastricht University Medical Center+, Maastricht, the Netherlands

**Keywords:** NK ‘helper’ cells, NK-DC interaction, PAMPs, Dendritic cells

## Abstract

**Background:**

Besides their prominent role in the elimination of infected or malignantly transformed cells, natural killer (NK) cells serve as modulators of adaptive immune responses. Enhancing bidirectional crosstalk between NK cells and dendritic cells (DC) is considered a promising tool to potentiate cancer vaccines. We investigated to what extent direct sensing of viral and bacterial motifs by NK cells contributes to the response of inflammatory DC against the same pathogenic stimulus.

**Results:**

We demonstrated that sensing of bacterial and viral PAMPs by NK cells contributes to DC cytokine production via NK cell-derived soluble factors. This enhancement of DC cytokine production was dependent on the pattern recognition receptor (PRR) agonist but also on the cytokine environment in which NK cells recognized the pathogen, indicating the importance of accessory cell activation for this mechanism. We showed in blocking experiments that NK cell-mediated amplification of DC cytokine secretion is dependent on NK cell-derived IFN-γ irrespective of the PRR that is sensed by the NK cell.

**Conclusions:**

These findings illustrate the importance of bidirectional interaction between different PRR-expressing immune cells, which can have implications on the selection of adjuvants for vaccination strategies.

**Electronic supplementary material:**

The online version of this article (10.1186/s12865-018-0247-y) contains supplementary material, which is available to authorized users.

## Background

Natural killer (NK) cells are important players of the innate immune system and are well described for their role in controlling viral infections and limiting tumour outgrowth by recognizing and eliminating altered self-cells [1–3]. The activation of NK cells is controlled by a tight balance of activating and inhibitory receptors between the interacting cells according to ‘missing-self’ or ‘stress-induced self-recognition’-principle [4] and/or by the surrounding cytokine milieu [5, 6]. The importance of NK cells in host defence and control of infections is supported by several studies with NK cell-deficient mice or mice having an impaired NK cell function, in which infections cause an increased viral load and lead to a higher mortality as compared to control animals [7]. Moreover, patients with NK cell deficiencies have an increased susceptibility to recurrent viral infections (mainly herpes- and papillomavirus) [8–11]. Interestingly, NK cell-deficient mice also displayed a higher bacterial load [12–14].

The presence of viral or bacterial pathogens is sensed by pattern recognition receptors (PRR). These receptors recognize conserved microbial structures, the so-called pathogen-associated molecular patterns (PAMPs). A variety of immune cells, such as antigen-presenting cells (e.g. dendritic cells; DC) express a specific repertoire of these PRR allowing a coordinated response of the different immune effectors cells against a particular pathogen [15–17]. Likewise, PAMP-matured DC have the capacity to activate NK cells by soluble as well as contact-dependent factors. This indirect sensing of pathogens includes enhancement of proliferation and survival as well as increased cytotoxic potential and cytokine secretion [18–21]. Moreover, several studies revealed that NK cells also express a diverse repertoire of PRR potentially allowing direct sensing of pathogens. This repertoire includes expression of all Toll-like receptors (TLR) 1–10 [22–26] as well as members of other PRR families: RIG1, NOD2, NRLP3, and MDA5 [27–29]. Various authors showed the functional importance of PAMP recognition with respect to the induction of cytotoxicity. Triggering of TLR2, 7, 8, and 9 enhances NK cell cytolytic potential against various tumour cell lines [22, 30–32]. Furthermore, TLR ligation can also influence the capacity of NK cells to edit the DC repertoire by eliminating suppressive immature DC (iDC) as well as the induction of pro-inflammatory cytokines [32]. Thus, direct PAMP-sensing by NK cells is not only influencing direct elimination of altered cells but can also modulate adaptive immune responses.

Another mechanism has been described by Maillard et al. who showed that NK cells can modulate immune responses [21]. Besides their important cytotoxic role, NK cells have also been ascribed a helper function. These helper NK cells have a DC activating capacity and induce type-1 polarizing DC. These DC are in turn able to produce high amounts of pro-inflammatory cytokines and to enhance Th1 and CTL responses [20, 21, 33, 34]. We have previously shown that a subset of NK cells is capable of producing IFN-γ after stimulation with DC-derived supernatant and identified the functional coupling of the potency of the NK cell helper response and Th1 polarization from the perspective of the DC [35]. The induction of these NK helper cells in vitro has been achieved by pro-inflammatory cytokines. Whether this helper activity of NK cells also gets induced upon direct sensing of pathogens remains understudied. Several studies show that direct PAMP triggering of NK cells can also lead to NK cell-derived cytokine production [22, 30–32]. A recent study by Wong et al. [36] showed that the addition of a TLR3 trigger to a NK-DC co-culture led to an enhanced DC maturation with respect to co-stimulatory molecule expression and IL-12p70 production as well as melanoma-specific CTL responses. It remains unclear whether this effect is influenced by the NK-DC crosstalk and whether the engagement of other PRR can induce a similar response. Further investigation on the role of PRR engagement by NK cells as an alternative activation pathway on the onset of an immune response is needed.

In the current study, we aimed to investigate in more detail whether the direct recognition of specific viral and bacterial PAMPs by NK cells contributes to an increased activation of mo-DC exposed to the same trigger during the initial phase of DC maturation. Therefore, we first studied the capacity of NK cells to respond to diverse viral and bacterial PAMPs. Furthermore, we aimed at identifying by virtue of which cytokines NK cells induce increased DC cytokine secretion and whether this mechanism differs between viral and bacterial activated NK cells.

## Methods

### Generation of DC

Leukapheresis products obtained from healthy volunteers were used to isolate the monocytes as previously described [37]; this study was approved by the local Medical Ethics Committee of Maastricht University Medical Center, the Netherlands (MEC azM/UM; MEC 08-2-120) and written informed consent was obtained from all participating healthy volunteers. Monocytes were differentiated in serum-free AIM-V^®^ medium (Life technologies, Carlsbad, CA, USA) supplemented with GM-CSF (400 U/ml; Berlex Laboratories Inc., Montville, NJ, USA) and IL-4 (2000 U/ml; Miltenyi Biotech GmbH, Bergisch Gladbach, Germany) at a density of 2 × 10^6^ cells/ml. After 7 days, iDC were harvested and frozen or immediately processed in *NK cell-induced DC maturation* assays.

### Flow cytometry

All antibodies used to determine NK cell purities as well as the surface marker expression of NK cells and DC were purchased from BD Biosciences (Franklin Lakes, NJ, USA). Antibodies were used, titrated to their optimal concentration, either as fluorescein isothiocyanate (FITC), phycoerythrin (PE), peridinin chlorophyll protein (PerCP), allophyocyanin (APC), allophyocyanin H7 (APC-H7), Horizon 450 or Pe-Cy7. Discrimination between dead and living cells was made based on LIVE/DEAD^®^ Fixable Dead Cell staining (Aqua stain; Life Technologies). Analysis were performed with BD FACS Canto II™ and analysed by BD FACSDiva™ Software v6.1.2 (BD Biosciences).

### NK cell isolation

NK cells were isolated from buffy coats or fresh peripheral blood-derived PBMC by negative immunomagnetic cell separation (Miltenyi Biotech) according to the manufacturer’s instructions. Blood was obtained from Sanquin blood bank Maastricht, the Netherlands (project 2000-03AZM) from healthy donors after informed consent. Isolated NK cells routinely exceeded 95% CD56^+^CD3^−^ (96.8% ± 0.87; containing ≤0.1% CD3^+^ cells, ≤ 0.1 CD19^+^ cells, and ≤0.5% CD56^−^CD16^−^ cells) as assessed by flow cytometry. The gating strategy is shown in Additional file 1: Figure S1.

### Activation of NK cells by PAMPs

For activation assays, we used CD56^+^CD3^−^ NK cells as in reports on the IFN-γ-secreting NK cell populations both CD56^bright^ and CD56^dim^ subsets have been shown to produce IFN-γ [20, 21, 38]. Freshly isolated NK cells were activated overnight in round-bottom 96-well plates (2.5 × 10^5^ cells/well) in serum-free AIM-V^®^ medium supplemented with various PAMPs and if indicated in the figure legends supplemented with different combinations of cytokines: IL-2 (1000 U/ml; Proleukin, Novartis, Basel, Switzerland); IL-2 and IL-18 (100 ng/ml; MBL International cooperation, Woburn, MA, USA); IL-12 (10 ng/ml; R&D systems, Minneapolis, MN, USA), IL-15 (20 ng/ml; R&D systems) and IL-18. The following PAMPs were used in this study: poly(I:C)HMW (50 μg/ml), poly(I:C)LMW (100 μg/ml), imiquimod (5 μg/ml), gardiquimod (5 μg/ml), CL075 (5 μg/ml), R848 (5 μg/ml), ssPolyU (5 μg/ml), ssRNA40 (5 μg/ml), Pam3CSK4 (5 μg/ml), HKLM (10^8^ cells/ml), FSL-1 (1 μg/ml), LPS (20 μg/ml), flagellin (10 μg/ml; all purchased from InvivoGen, Toulouse, France), and FMKp (10 μg/ml; Pierre Fabre Laboratories, Boulogne-Billancourt, France). The PAMP concentrations used to activate NK cells correspond to the working concentrations indicated by InvivoGen or by other publications. FMKp has been titrated as described in Oth et al. [35]. As control ‘supernatants’, additional wells on the same plate containing medium and PAMPs with or without cytokine cocktails were incubated overnight. After 16-18 h of incubation, cell-free supernatants and control supernatants were harvested and used to mature iDC. Additionally, NK cell-derived cytokine and chemokine profiles were determined. The remaining cells were stained for various cell surface markers and were analysed by flow cytometry.

### DC maturation induced by NK cell-derived soluble factors

Supernatants of activated NK cells and control supernatants (medium containing same concentrations of PAMPs as initially used to activate NK cells with or without cytokines stored overnight in the incubator without the presence of NK cells) were transferred into flat-bottom 96-well plates supplemented with IL-4 (500 U/ml) and GM-CSF (500 U/ml). Both conditions NK cell-derived supernatant and control ‘supernatant’ contained PAMPs. To each well, 2.5 × 10^5^ iDC were added. After 48 h of maturation, the supernatant was harvested and analysed for DC-derived cytokines and chemokines. Additionally, DC were harvested, stained for phenotypic surface markers and analysed by flow cytometry.

### Blocking studies

Blocking studies were performed with cell-free supernatants obtained from freshly isolated NK cells activated overnight in serum-free AIM-V^®^ medium and IL-2 (1.000 U/ml) supplemented with FMKp (10 μg/ml) or poly(I:C)HMW (50 μg/ml). The receptor-blocking was performed by pre-incubating iDC with blocking antibodies for 20 min before their addition into flat-bottom 96-well plates containing the cell-free NK cell supernatant supplemented with IL-4 (500 U/ml) and GM-CSF (500 U/ml). The following receptor blocking antibodies were used: IFNGR1 (20 μg/ml), TNFR1 (20 μg/ml), TNFR2 (20 μg/ml), or isotype control (all purchased from R&D systems). The blocking of the cytokines (IFN-γ and TNF-α) in the NK cell-derived supernatants was performed by pre-incubating the supernatants with anti-TNF-α (20 μg/ml; BD) or anti-IFN-γ (10 μg/ml; BD) before adding the iDC. As reference value, iDC were incubated with NK cell-derived supernatant in the absence of blocking agents. As a negative control, iDC were incubated with medium supplemented with FMKp or poly(I:C) and IL-2 (control ‘supernatant’). After 48 h of maturation, the supernatant was harvested to determine the DC cytokine and chemokine profiles.

### Supplementation studies

iDC were matured in 48 well plates (2.5 × 10^5^ cells/well) in serum-free AIM-V^®^ in the presence of IL-4 (500 U/ml) and GM-CSF (500 U/ml) supplemented with different concentrations of rhIFN-γ (0–50.000 U/ml; R&D systems) or rhTNF-α (0–10.000 U/ml; Life Technologies). The supernatant was harvested after 48 h of maturation and cytokines were quantified by cytometric bead array (CBA; see below).

### Cytokine detection

Quantification of secreted DC- and NK cell-derived pro-inflammatory cytokine and chemokine profiles was performed by CBA flex set assay (BD Biosciences) according to the manufacturer’s instructions. Measurements were performed with BD FACS Canto II™ and analysed by BD FACSDiva™ Software v6.1.2 and FCAP array™ analysis software (version 1.0.1; Soft Flow Inc., St. Louis Park, MN, USA).

### Statistical analyses

Statistical analyses were determined by Kruskal-Wallis (NK cell analyses), Mann-Whitney U test (DC analyses) or by paired T-test (blocking studies) and the correlation was tested by nonparametric Spearman correlation; * *P* ≤ 0.05, ** *P* ≤ 0.01, *** *P* ≤ 0.001, **** *P* ≤ 0.0001. Data were analysed using GraphPad Prism Software (version 6; GraphPad Software, San Diego, CA, USA).

## Results

### Viral PAMP-recognition by NK cells induces their activation in the presence of IL-2

**Fig. 1:**
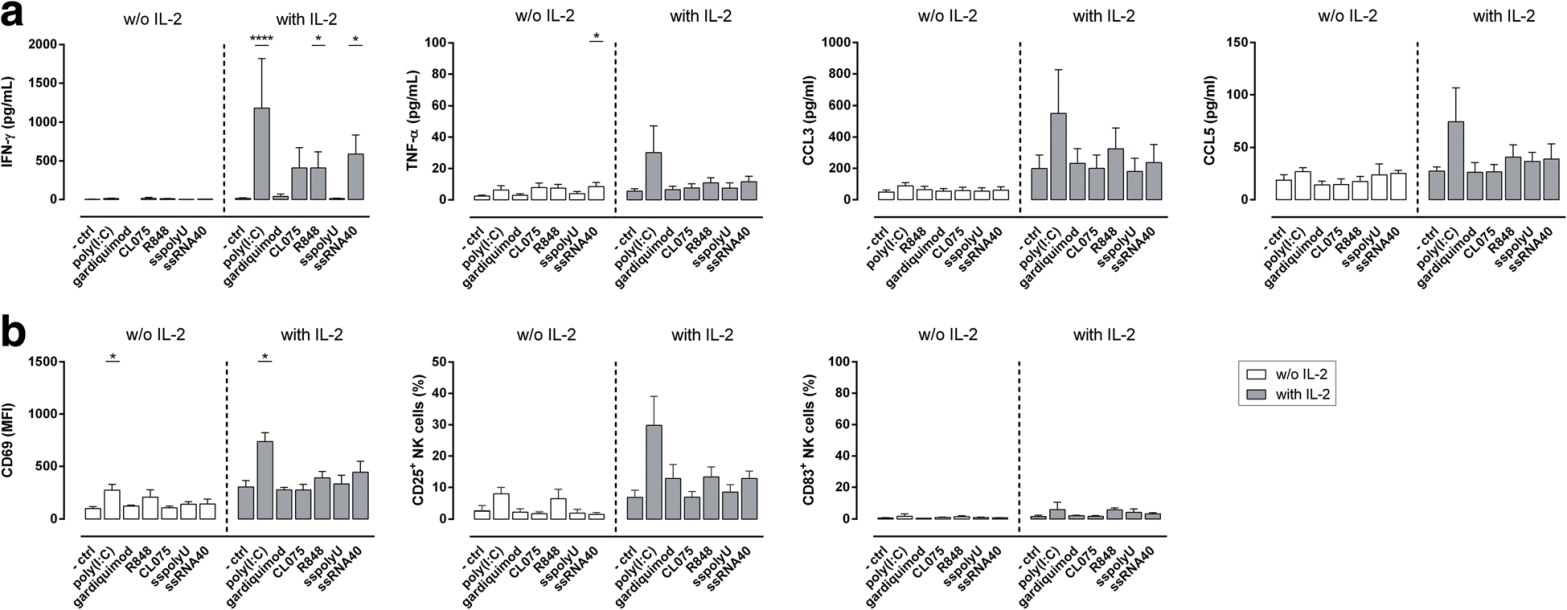
Sensing of viral PAMPs triggers NK cell activation and cytokine secretion. NK cells were activated overnight in round-bottom 96-well plates (2.5 × 10^5^ cells/well) with viral PAMPs in the absence (white bars) or presence (gray bars) of IL-2 in serum-free medium. As a negative control, NK cells were incubated with medium alone. **a** After 16-18 h of incubation IFN-γ, TNF-α, CCL3, and CCL5 were determined in the culture supernatant by CBA. **b** After 16-18 h of incubation cells were harvested and stained for CD69, CD25, and CD83. Expression of these markers was analysed by flow cytometry. All data are presented as mean + SEM and are representative of at least 3 independent experiments (3 ≤ *n* ≤ 12). After an initial screening of at least 3 independent experiments for each ligand, future experiments were peformed mainly with the ligands showing a positive effect on NK cell activation. Kruskal-Wallis test significance as compared to negative control with and without IL-2, respectively. * *P*≤0.05, **** *P*≤0.0001

To evaluate and confirm whether NK cells recognize and get activated after engagement of different viral PAMPs (triggering various TLR), we incubated NK cells with different viral TLR ligands: poly(I:C) (TLR3), gardiquimod (TLR7), CL075 and R848 (both TLR7/8), ssRNA40 and sspolyU (both TLR8). In the NK cell-derived supernatant, we measured the amount of secreted cytokines and chemokines by cytometric bead array (CBA). After viral TLR ligation, the amount of IFN-γ and TNF-α detected in NK cell-derived supernatant was minimal and did not differ between the different viral PAMPs (Fig. 1a). Furthermore, we determined the production of the chemokines involved in attraction and enhanced cytotoxity of NK cells [39, 40] as well as the recruitment of iDC [34]: CCL3 and CCL5. The chemokine production was low and no differences were observed between the different triggers (Fig. 1a). The expression of activation markers CD69 and CD25 was upregulated by NK cells activated with the TLR3 trigger poly(I:C) and the TLR7/8 trigger R848 (Fig. 1b).

Since it has been previously reported that NK cells get more efficiently activated by the combination of two signals [33], we investigated whether the addition of IL-2 to the viral PAMPs during overnight incubation enhances PAMP-induced NK cell activation. The presence of IL-2 enhanced CCL3 secretion, as well as CD69 and CD25 expression levels in the control condition. By adding IL-2, we also observed differences in the ability of NK cells to sense viral PAMPs and to induce cytokine and chemokine secretion as well as upregulation of cell surface markers. Only triggering TLR3 by poly(I:C) in combination with IL-2, enhanced all the analysed parameters and was superior in cytokine secretion and surface marker expression compared to all other triggers. R848 and ssRNA40 induced a significant increase in NK cell-derived IFN-γ (Fig. 1a, b). The limited response towards TLR7 trigger gardiquimod was observed as well as for another TLR7 PAMP, imiquimod (data not shown). Notably, whereas of the two TLR8 triggers ssRNA40 and ssPolyU only ssRN40 induced IFN-γ production by NK cells, the induction of the other parameters was comparable between these two PAMPs.

These data show that NK cells are able to respond to different viral triggers, however, for efficient sensing NK cells require co-activating factors (e.g. IL-2) to induce potent cytokine secretion. Additionally, we observed that the different TLR-triggering PAMPs, but also PAMPs triggering the same TLR (e.g. TLR8) differ in their potency to induce an NK cell response. Besides TLR3 triggers which showed an NK cell stimulatory effect, also other viral TLR triggers led to an efficient IFN-γ production such as TLR7/8 triggers.

### Bacterial PAMP-recognition by NK cells induces their activation in the presence of IL-2

**Fig. 2:**
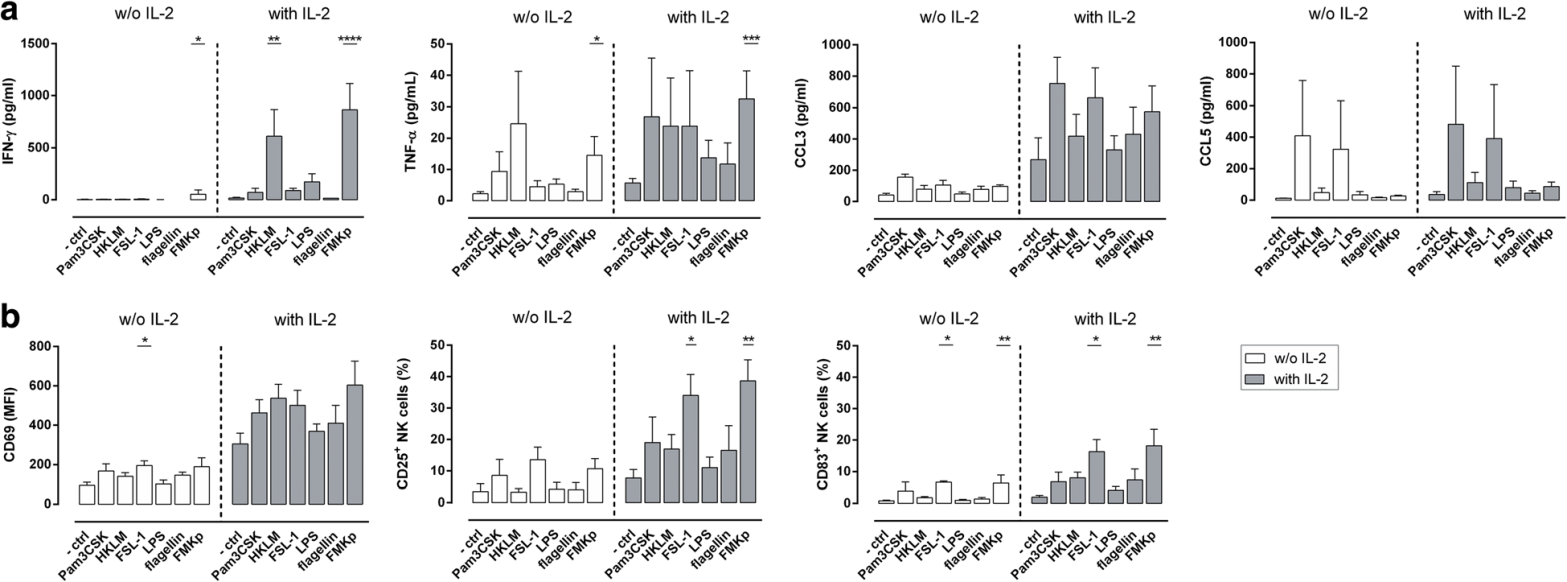
Sensing of bacterial PAMPs triggers NK cell activation and cytokine secretion. NK cells (2.5 × 10^5^ cells/well) were activated overnight in round-bottom 96-well plates with various bacterial PAMPs in the absence (white bars) or presence (gray bars) of IL-2 in serum-free medium. As a negative control, NK cells were incubated with medium alone. **a** After 16-18 h of incubation IFN-γ, TNF-α, CCL-3, and CCL5 were determined in the culture supernatant by CBA. **b** After 16-18 h of incubation cells were harvested and stained for CD69, CD25, and CD83. Expression of these markers was analysed by flow cytometry. All data are presented as mean + SEM and are representative of at least 3 independent experiments (3 ≤ n ≤ 12). Kruskal-Wallis test significance as compared to negative control with and without IL-2, respectively. * *P*≤0.05, ** *P*≤0.01, *** *P*≤0.001, **** *P*≤0.0001

In addition to viral PRR, NK cells are also equipped with TLR sensing bacterial PAMPs. Therefore, we investigated whether bacterial triggers activate and induce cytokine production by NK cells. We incubated NK cells with various bacterial PAMPs triggering diverse TLR: Pam3CSK4 (TLR1/2), HKLM (TLR2), FSL-1 (TLR2/6), LPS (TLR4), flagellin (TLR5) and FMKp (a lysate containing membrane fragments of *Klebsiella pneumoniae*, triggering multiple PRR). In the absence of IL-2, only FMKp-activated NK cells showed significant increased IFN-γ secretion levels as compared to control. This was even further increased in the presence of IL-2 (Fig. 2a). In the presence of IL-2, HKLM and FMKp induced superior IFN-γ secretion levels as compared to the control. Overall, the TNF-α secretion was low; only FMKp-triggered NK cells showed superior TNF-α levels as compared to the control (in absence and presence of IL-2). Moreover, bacterial TLR-triggering induced NK cell-derived chemokine production CCL3 and CCL5 in all the conditions. No significant differences were seen between control condition and various bacterial PAMP conditions. (Fig. 2a). In addition, we analysed surface marker expression of the differently activated NK cells. IL-2 had a positive effect on the cell surface marker expression. No differences were observed in the expression levels of CD69 between the different bacterial PAMP-triggered NK cells. FMKp-activated NK cells showed increased CD25^+^ and CD83^+^ subpopulations in presence of IL-2 (Fig. 2b). In presence or absence of IL-2, FSL-1 also induced a high percentage of CD25^+^ and CD83^+^ NK cells, comparable to FMKp-activated NK cells.

Thus, NK cells are able to respond to different bacterial triggers, which requires similarly to  viral stimuli additional co-activating factors for efficient sensing by NK cells.

### PAMP-activated NK cells provide help for DC maturation

**Fig. 3:**
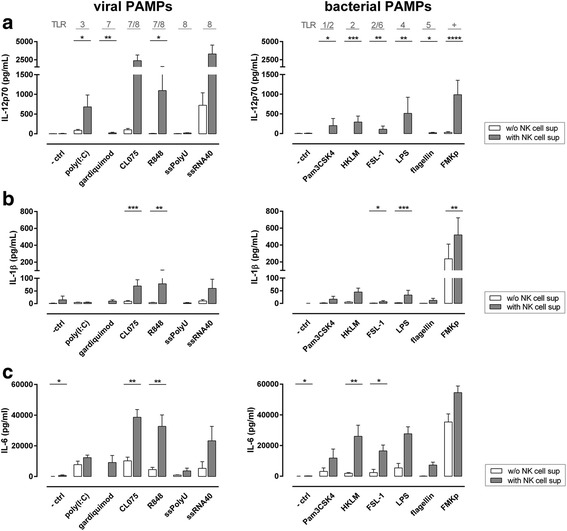
Soluble factors derived from viral and bacterial-triggered NK cells increase the pro-inflammatory DC cytokine profile. NK cells (2.5 × 10^5^ cells/well) were activated overnight in round-bottom 96-well plates with various viral and bacterial PAMPs (indicated on x-axis) in the presence of IL-2. As control, viral and bacterial PAMPs were stored overnight in the incubator without the presence of NK cells. iDC were matured in these cell-free NK cell-derived supernatants (gray bars) or control supernatants (white bars) supplemented with IL-4 (500 U/ml) and GM-CSF (500 U/ml). After 48 h, DC-derived cytokines were determined in the culture supernatant by CBA. **a** IL-12p70 production. Mean + SEM of *n* ≥ 9 independent experiments is shown. **b** IL-1β production. Mean + SEM of *n* ≥ 8 independent experiments is shown. **c** IL-6 production. Mean + SEM of *n* ≥ 3 independent experiments is shown. Mann-Whitney U test comparing absence and presence of NK cell-derived supernatant (white vs. gray bars). * *P* ≤ 0.05, ** *P* ≤ 0.01, *** *P* ≤ 0.001, **** *P* ≤ 0.0001

We showed that NK cells get activated by both viral and bacterial PAMPs triggering diverse TLR in the presence of IL-2. Next, we studied whether soluble factors derived of these PAMP-activated NK cells can modulate immune responses by providing help for DC maturation. Monocyte-derived DC have been described to express TLR1–8 and TLR10 [24, 41–44]. We investigated whether NK cell-derived soluble factors are facilitating DC pro-inflammatory cytokine responses. iDC were matured in the absence (control ‘supernatant’ containing PAMPs) or presence of NK cell-conditioned medium derived from NK cells activated with various PAMPs in the presence of IL-2. In a previous study, we showed that DC-derived IL-12p70 production positively correlates with their capacity to activate NK cells and to induce Th1 responses [35] and thus playing a crucial role in coordinating potent type 1 immune responses. Therefore, we first analysed the capacity of NK cells to induce DC-derived IL-12 production. DC matured with the viral TLR3 and 7/8 triggers displayed enhanced IL-12p70 production in the presence of NK cell supernatant. A marginal effect was observed with TLR7 triggers gardiquimod (Fig. 3a) and imiquimod (data not shown). Notably, only after stimulation with one of the two TLR8 triggers, ssRNA40, the DC-derived IL-12p70 production was enhanced in the presence of NK cell-conditioned medium. DC matured in the presence of conditioned medium derived from bacterial PAMP-triggered NK cells displayed all an enhanced capacity to produce IL-12p70 as compared to DC matured with the respective PAMPs alone; the highest increase was observed with FMKp (Fig. 3a).

We further analysed the capacity of DC to produce IL-1β (Fig. 3b). The production was enhanced for both viral 7/8 triggers, CL075 and R848, and ssRNA40 in the presence of NK cell-derived supernatant. The secretion of IL-1β was enhanced in all the conditions for the bacterial PAMPs, however, the production levels were low compared to FMKp (Fig. 3b). The secretion of IL-6 was enhanced in all conditions in the presence of NK cell-conditioned medium (Fig. 3c). The IL-10 producing capacity of DC was not influenced by the presence of NK cell-conditioned medium (data not shown).

**Fig. 4:**
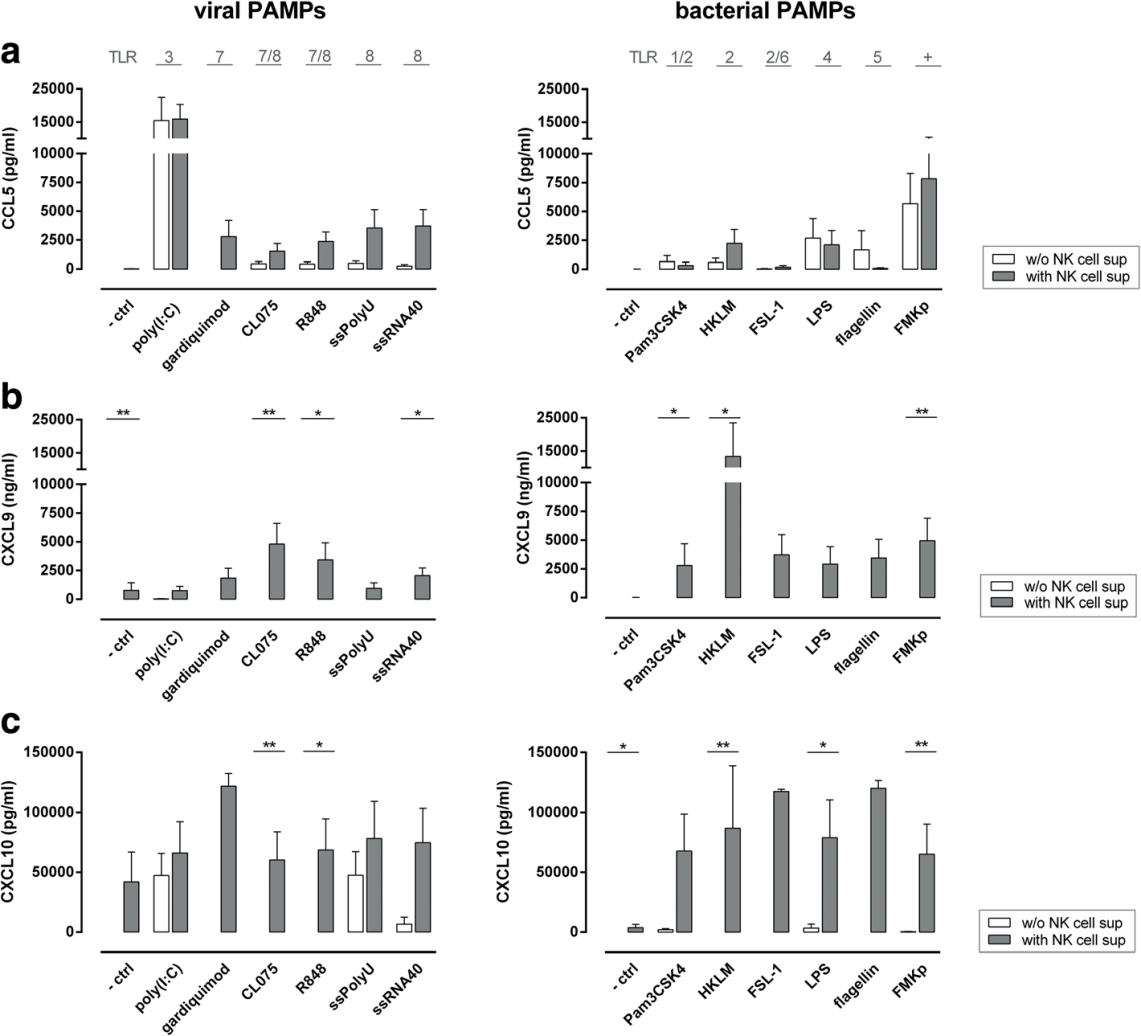
Soluble factors derived from viral and bacterial-triggered NK cells increase the pro-inflammatory DC chemokine profile. NK cells (2.5 × 10^5^ cells/well) were activated overnight in round-bottom 96-well plates with various viral and bacterial PAMPs (indicated on x-axis) in the presence of IL-2. As control, viral and bacterial PAMPs were stored overnight in the incubator without the presence of NK cells. iDC were matured in these cell-free NK cell-derived supernatants (gray bars) or control supernatants (white bars) supplemented with IL-4 (500 U/ml) and GM-CSF (500 U/ml). After 48 h, DC-derived chemokines were determined in the culture supernatant by CBA. **a** CCL5 production. **b** CXCL9 production. **c** CXCL10 production. Mean + SEM of n ≥ 3 independent experiments is shown. Mann-Whitney U test comparing absence and presence of NK cell-derived supernatant (white vs. gray bars). * *P*≤0.05, ** *P*≤0.01

We next determined the secretion of the chemokine CCL5 and of the two CD8^+^ T cell-recruiting chemokines CXCL9 and CXCL10. In the absence of NK cell-derived supernatant, FMKp, LPS, flagellin, and poly(I:C) did induce CCL5 secretion (Fig. 4a). The production of CCL5 was enhanced for all viral triggers in the presence of NK cell supernatant except poly(I:C) and for the bacterial triggers HKLM and FMKp. Both virally and bacterially activated NK cell supernatants enhanced the capacity of DC to produce CXCL9 and CXCL10 (Fig. 4b,c). In the absence of NK cell supernatant only some of the viral triggers (poly(I:C), ssPolyU and ssRNA40) directly induced CXCL10 production by DC (Fig. 4c).

### The potency of the NK cell accessory effect after pathogen sensing is determined by the cytokine milieu

It is well established that the activation of NK cells is influenced by the local cytokine environment [5, 21, 45]. We studied whether the cytokine milieu in which NK cells recognize the different PAMPs would also enhance the capacity of NK cells to amplify the pro-inflammatory cytokine profile of DC. We studied the effect of cytokine environment with a bacterial trigger. To this end, we activated NK cells in the presence of FMKp and different combinations of cytokine cocktails, previously shown to be efficient inducers of NK cell activation on their own [21, 46, 47].

**Fig. 5:**
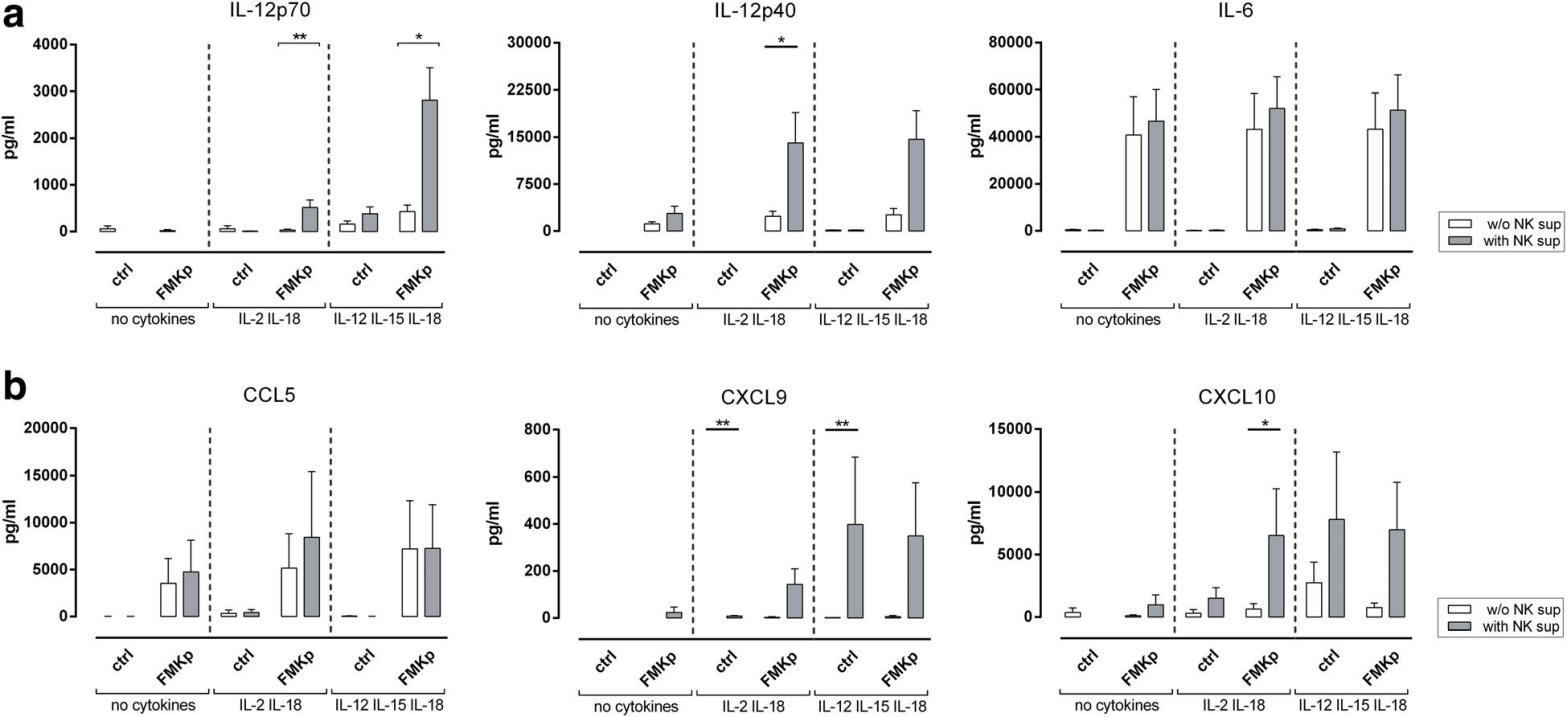
Influence of cytokine milieu. NK cells were activated overnight in serum-free medium in the absence or presence of FMKp with or without different cytokine combinations (no cytokines; IL-2/IL-18; IL-12/IL-15/IL-18). iDC were matured in presence or absence of these NK cell-derived supernatants for 48 h. DC-derived cytokine and chemokine secretion was determined in cell-free supernanant by CBA. **a** Cytokine profile. **b** Chemokine profile. Data are shown as mean + SEM of *n* ≥ 4 independent experiments. Mann-Whitney U test comparing absence and presence of NK cell-derived supernatant (white vs. gray bars). * *P*≤0.05, ** *P*≤0.01

The DC-derived secretion of IL-12p70 was enhanced by the cytokine milieu in which NK cells encountered FMKp; in presence of IL-2 and IL-18 the secretion of IL-12p70 was significantly increased in the presence of NK cell-conditioned medium compared with control. This was even further increased when NK cells encountered FMKp in the presence of IL-12, IL-15 and IL-18. Additionally, also the production of IL-12p40 and the T cell-attracting chemokines CXCL9 and CXCL10 was enhanced in the presence of NK cell-conditioned medium. No additive effect was observed for the secretion of IL-6 and CCL5 (Fig. 5). It should be noted that, as seen in Figs. 3c and 4a, FMKp-matured DC secreted IL-6 and CCL5 in the absence of NK cell-conditioned medium and cytokines, which is not further increased by means of NK cell help.

### IFN-γ-mediated NK cell help for enhanced DC cytokine and chemokine secretion

**Fig. 6:**
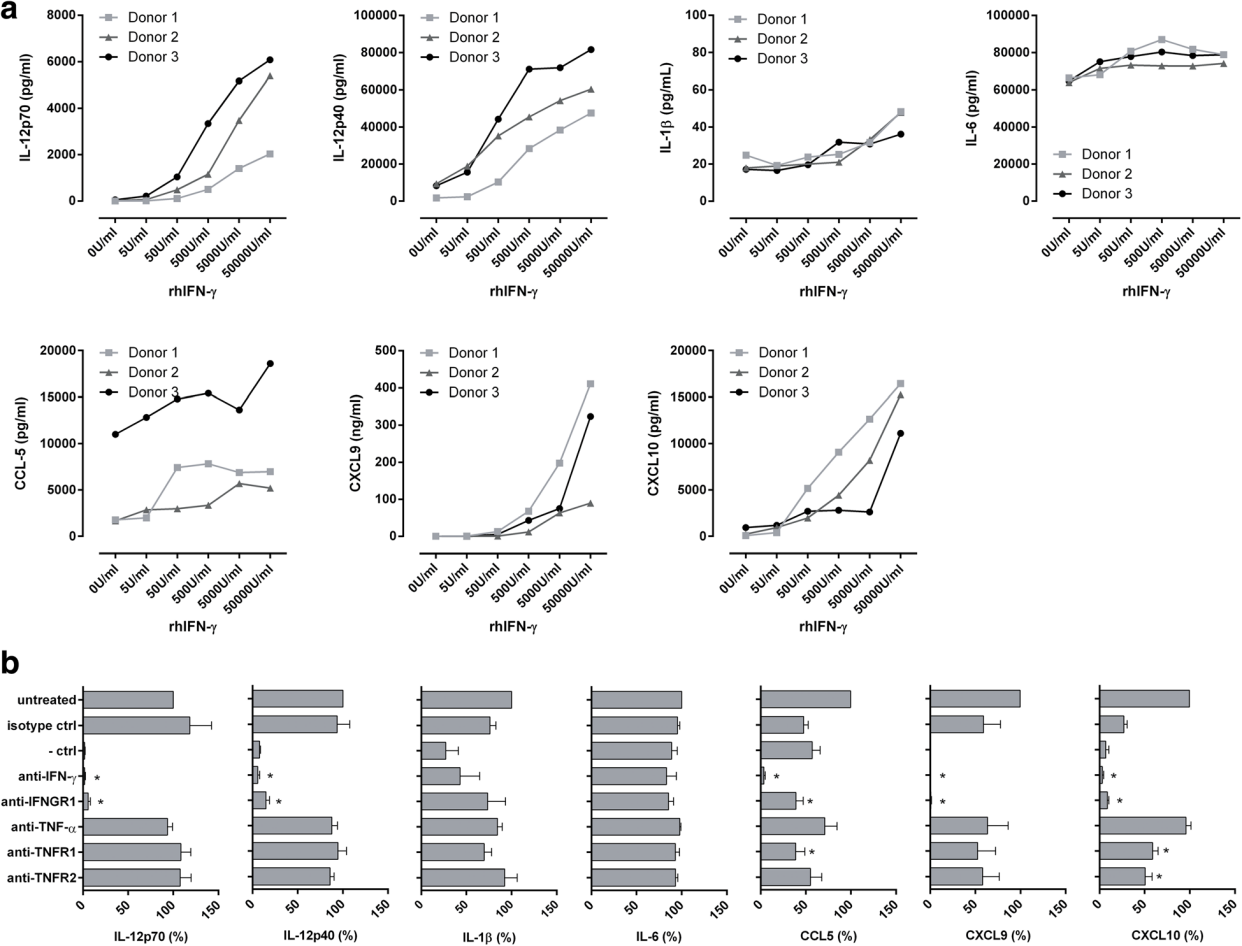
NK cell-derived IFN-γ but not TNF-α is necessary to modulate the pro-inflammatory DC cytokine profile. **a** Supplementation of rhIFN-γ during DC maturation and its effect on cytokine and chemokine secretion is shown. iDC were matured in serum-free medium supplemented with FMKp, IL-4 and GM-CSF in the presence of increasing concentrations of rhIFN-γ. Cytokine and chemokine profiles were determined by CBA in the culture supernatants after 48 h of maturation. The individual donors are shown. **b** Blocking of IFN-γ and TNF-α during DC maturation and its effect on cytokine and chemokine secretion is shown. iDC were matured in the cell-free supernatant derived from FMKp-activated NK cells in the presence of IL-2. As indicated on the y-axis, various blocking antibodies were added: anti-IFN-γ, anti-IFNGR1, anti-TNF-α, anti-TNFR1, and anti-TNFR2. iDC matured with NK cell-derived supernatant in the absence of blocking antibodies (untreated) were used as reference value and set at 100% capacity to produce a cytokine/chemokine (x-axis). The negative control represents the capacity of DC to produce the indicated cytokine upon maturation with FMKp in absence of NK cell- derived supernanant. Mean + SEM of 3 independent experiments is shown. Statistical analyses were performed on normalized data using paired t-test comparing untreated DC versus DC conditions containing blocking antibodies. Significance is indicated by *

IFN-γ as well as TNF-α have been described as factors involved in DC maturation [48]. We identified a significant positive correlation between the amounts of pro-inflammatory cytokine IFN-γ and, in parallel, TNF-α produced by the differently activated NK cells with their respective DC-derived IL-12p70 levels (data not shown). To elucidate whether both NK cell-derived IFN-γ and TNF-α play a role in the mediated effect on DC maturation, we performed supplementation studies with rhIFN-γ and rhTNF-α. The addition of increasing doses of IFN-γ to iDC in the presence of FMKp led to increased production of IL-12p70, IL-12p40, CXCL9 and CXCL10 by DC (Fig. 6a). In contrast, the effect of supplementation on IL-6, IL-1β and CCL5 production was marginal. The secretion of the anti-inflammatory cytokine IL-10 was not influenced (data not shown). Furthermore, the supplementation of IFN-γ to poly(I:C)-matured DC showed similar results as FMKp-matured DC (Additional file 2: Figure S2). However, no effect was observed by supplementing rhTNF-α during FMKP-DC maturation (Additional file 3: Figure S3).

To confirm these findings, we performed blocking studies. The supernatant of FMKp and IL-2 activated NK cells was added to iDC in the absence or presence of anti-IFN-γ or anti-TNF-α or presence of receptor blockers (anti-IFNGR1, anti-TNFR1, or anti-TNFR2). Blocking of IFN-γ by either capturing IFN-γ in the supernatant or IFNGR1 on the surface of iDC dramatically reduced the capacity of DC to produce IL-12p70 (remaining capacity by blocking with anti-IFN-γ: 1.67% ± 1.11), IL-12p40 (5.79% ± 2.09), CXCL9 (0.09% ± 0.07) and CXCL10 (3.12% ± 1.39) as compared to untreated DC matured in the presence of NK cell-conditioned medium (Fig. 6b). In line with the supplementation of rhIFN-γ, marginal or no effect was observed on the capacities of DC to secrete IL-1β, IL-6 and CCL5. Furthermore, blocking of TNF-α did not influence the cytokine secretion during the initial phase of DC maturation. The same effect was observed for DC which have been matured in the presence of poly(I:C)-activated NK cell-derived supernatant and blocking antibodies (data not shown) as well as for DC matured with cytokine-activated (IL-2 and IL-18) NK cell-derived supernatant (Additional file 4: Figure S4).

## Discussion

In the current study, we set out to investigate whether the direct sensing of viral and bacterial pathogens by NK cells would not only increase their cytotoxic potential as previously shown [22, 30–32], but also induce helper responses for DC maturation, as it has been previously shown for cytokine activated NK cells [21, 33, 34]. Of note, this study specifically aimed at identifying the amplifying effect of NK cell-derived soluble factors on DC maturation (encountering the same pathogenic stimuli). In our experimental set-up, the PAMP concentration may be lower in the NK cell-conditioned medium as compared to the control medium, because it is bound or internalized by the NK cells. As we have previously shown that DC-derived IL-12p70 production is PAMP dose-dependent [35], a reduced effect on the dendritic cell response could be expected. Thus, the experimental design only allows the detection of an amplifying effect of NK cells but not an inhibiting effect of the NK cell-conditioned medium on the dendritic cell maturation. We identified several viral and bacterial PAMPs able to induce functional activation of NK cells (TLR1/2, TLR2, TLR2/6, TLR 3, TLR4, TLR7/8, and TLR8 triggers) with respect to pro-inflammatory cytokine and chemokine secretion (IFN-γ, TNF-α, CCL3, CCL5) and upregulation of activation markers (CD69, CD25, CD83). In contrast, NK cells showed limited responses towards TLR5 trigger flagellin and TLR7 triggers imiquimod and gardiquimod. The amplification of DC responsiveness was dependent on the local cytokine environment in which the NK cells encountered the pathogen. We showed that soluble factors released by PAMP-triggered NK cells are responsible for the amplification of the pro-inflammatory cytokine response of moDC and we identified IFN-γ as a crucial cytokine for the amplification of the initial DC response.

Previously, a study of Wong et al. [36] addressed the effect of poly(I:C) on DC maturation during NK-DC co-culture. The addition of poly(I:C), IL-18, and IFN-α to the NK-DC co-culture significantly enhanced stable type 1 polarized DC producing high amounts of IL-12. In our study, we extended these findings and addressed the question whether recognition of other viral and bacterial PAMPs by NK cells facilitates DC maturation. In a contact-independent set-up, we revealed that the helper activity of both viral and bacterial triggered NK cells on DC cytokine and chemokine production (IL-12p70, IL-12p40, CXCL9 and CXCL10) was dependent on IFN-γ. We revealed that the presence of IFN-γ during DC maturation is crucial for efficient DC-derived cytokine secretion. The secretion of IL-6, IL-1β and CCL5 was independent of IFN-γ. Interestingly, Mailliard et al. [33] have demonstrated convincingly that NK cell-derived TNF-α also contributes to the DC-derived cytokine secretion. Their demonstration of the importance of TNF-α is not in contradiction to our study as both studies address a different phase of DC biology. Our study aimed at identifying the initial phase of DC maturation following recognition of the pathogen, whereas Mailliard and colleagues addressed the importance of these cytokines in the second release of IL-12p70 after the DC-T cell contact (upon CD40L stimulation).

The influence of the cytokine milieu on the strength of PRR-induced NK cell activation and the resulting helper properties suggest that the coordinated immune response induced by a particular pathogen can be influenced by all PRR-expressing immune cells. As such, an accessory cell can be triggered by a specific PAMP to release a defined set of pro-inflammatory cytokines. In turn, these cytokines, e.g. IL-12, lead to an enhanced PAMP recognition by NK cells and their activation. Activated NK cells control the immune response by direct killing of infected cells, Treg or by DC editing as well as providing cytokines and chemokines. TLR-activated NK cells are able to secrete CCL3, CCL4 and CCL5 to recruit iDC [34] as well as IFN-γ by which the DC maturation and Th1 polarization are further enhanced. Furthermore, those NK cells induce CXCL10 and CXCL9 producing CD8^+^ T effector cell recruiting DC, which has been previously only observed with IL-18 primed NK cells [34]. The specific response against a pathogenic insult has thus different checkpoints, which act as regulators or amplifiers. We and others [22, 28] showed that the recognition of individual TLR by NK cells as well as the subsequent NK cell helper activity mostly requires the presence of accessory cytokines. Thus, we propose that the direct sensing of pathogens by NK cells functions as an amplifier of the initiated immune responses, rather than as initiator of pathogen-specific immune responses. The accessory cells determine the local cytokine milieu and thus regulate the strength of NK cell activation and subsequently the kinetics of the NK helper cell-mediated augmentation of immune responses.

The presence of accessory cytokines was of crucial importance for an optimal sensing of both bacterial and viral PAMPs and induction of NK helper cell properties. Here we show that the addition of IL-2, the combination of IL-18 and IL-2, or IL-12, IL-15 and IL-18 influence activation of NK cells and their helper activities, which were marginal in the absence of these cytokines. NK cells briefly pre-activated by this combination of cytokines (IL-12, IL-15 and IL-18) were previously shown to have a sustained effector function in tumour mouse models and an enhanced proliferative capacity as well as an increased IFN-γ response over time in human in vitro studies [46, 47]. We show that by adding FMKp, the helper capacity of these NK cells to induce DC maturation can be further improved (up to 12-fold increase in IL-12p70 secretion).

We previously showed that DC-derived IL-12p70 was positively correlated with the capacity of DC to induce Th1 responses and activate IFN-γ-producing NK helper cells [35]. NK cell-derived IFN-γ production induced by DC-derived soluble factors was dependent on IL-12p70 and the IFN-γ-producing NK cells were not confined to the CD56^bright^ subset. Here, we showed that IFN-γ producing NK helper cells can also be generated in the presence of a TLR trigger and an accessory cell-derived cytokine, e.g. IL-2. This offers the possibility that cell-independent NK helper cell activation in vivo may occur via two different mechanisms, one being dependent on 2 signals of which IL-12 is indispensable in the presence of only soluble factors and the other independent of IL-12 in the presence of pathogenic triggers.

Among the different viral triggers tested in this study, poly(I:C) (TLR3 trigger), R848 and CL075 (both TLR7/8 triggers) activated NK cells most efficiently including the induction of helper properties. Previous studies showed that sensing of these triggers stimulated cytolytic capacities [22, 32]. Thus, sensing of these PAMPs potentially induces both the helper and killer capacities of NK cells. Nonetheless, the extent of the induction of both programs may be dependent on the local cytokine environment in vivo. Notably, whereas both TLR7/8 triggers induce NK cell helper properties, this effect was only observed for one of the two TLR8 triggers and for none of the TLR7 triggers. Even though, ssPolyU and ssRNA both induced NK cell activation to a similar extent (activation marker upregulation and chemokine secretion), ssPolyU failed at inducing IFN-γ production explaining the differences observed in providing help for DC-induced cytokine production. Activating human NK cells with imiquimod has previously been shown to enhance cytotoxicity of NK cells, but not IFN-γ secretion [25]. Furthermore, another study showed that both gardiquimod and imiquimod could enhance NK cell proliferation as well as cytotoxicity in mice [49]. Yet another study observed enhanced IFN-γ secretion after activation of NK cells with the TLR7 trigger loxoribine [30]. Different from gardiquimod and imiquimod, which are both imidazoquinoline compounds, loxorobine is a guanosine analogue. Apparently, depending on the choice of compound to trigger TLR7, the NK cell helper program will or will not get induced.

Previously, we showed indirect activation of NK cells via DC that were matured in the presence of bacterial pathogens [20]. The current study confirms the finding of other groups that NK cells have the capacity to directly respond to bacterial pathogens. In addition to these studies, we showed that the direct sensing of bacterial pathogens by NK cells also enhances NK cell-mediated help for DC maturation and is not only limited to the induction of their cytotoxic capacity. All the bacterial PAMP-triggered NK cells enhanced DC-derived IL-12 production, however, the effect induced by TLR5 activated NK cells was minor. Possibly, the helper function may be a more important mechanism in response against bacterial pathogens than cytotoxic function. The strongest response against bacterial PAMPs was induced by FMKp. In a previous study, we observed IL-18 production by FMKp-matured DC [20]. Together with the highest IL-1β production detected in the current study, this suggests the involvement of inflammasome activation. Hence, a stronger response may be linked to simultaneous engagement of multiple PRR, as the bacterial lysate FMKp possibly triggers a range of different PRR. Arguably, autonomous activation of NK cells by PRR occurs more easily upon multiple receptor stimulation as e.g. shown for HCMV infection or tuberculosis [50, 51].

TLR triggers do not only lead to indirect NK cell activation via DC-dependent mechanisms as previously shown [18–21] but as illustrated in this study, NK cells can also be directly activated by TLR triggers. For DC vaccination the bidirectional NK-DC crosstalk is important for efficient immune cell activation [20, 52]. With the findings described in this study and others [36], one could argue that DC used for vaccination strategies should be matured in vitro in the presence of NK cells or NK cell-derived factors. Moreover, to ensure potent NK cell activation, there is rationale for safety trials in which co-administration of selected TLR triggers with DC-based vaccines is performed to ensure enhanced NK cell activation.

## Conclusion

Taken together these data show that NK cells, besides their crucial role in host defence for the elimination of virally infected or malignantly transformed self-cells, also have an important role in the amplification of adaptive immune responses. In the current study, we demonstrated that NK cells can sense various viral as well as bacterial PAMPs, which is enhanced in the presence of cytokines from accessory cells. TLR-induced NK helper cells can augment the pro-inflammatory phenotype of DC via the production of IFN-γ. The magnitude of this amplification depends on the dose of NK cell-derived IFN-γ and this dose is determined by the triggered PRR, the choice of ligand for a particular PRR, and the cytokine environment in which the NK cell recognizes the microbial pattern. This illustrates once more the importance of TLR network and how the invasion of pathogens regulates a specific tailored immune response. Moreover, this knowledge can be of importance to generate more potent and functional type 1-polarized cells in vitro and in vivo, e.g. for boosting vaccines.

## Additional files

Additional file 1: Figure S1. Flow cytometric analysis of NK cell purity. NK cells were gated in the FSC/SSC on the lymphocyte gate and dead cells were excluded by live/dead staining (7-AAD). Percentages of CD19^+^, CD3^+^, CD56^+^CD3^−^, CD56^−^CD16^+^ are indicated in the plots. (TIFF 274 kb)

Additional file 2: Figure S2. Supplementation of rhIFN-γ during poly(I:C)-DC maturation and its effect on cytokine and chemokine secretion**.** iDC were matured in serum-free medium supplemented with poly(I:C), IL-4, and GM-CSF in the presence of increasing concentrations of rhIFN-γ. Cytokine and chemokine profiles were determined in the culture supernatants after 48 h of maturation by CBA. Three individual donors are shown. (TIFF 1638 kb)

Additional file 3: Figure S3. Supplementation of rhTNF-α during FMKp-DC maturation and its effect on cytokine and chemokine secretion. iDC were matured in serum-free medium supplemented with FMKp, IL-4, and GM-CSF in the presence of increasing concentrations of rhTNF-α. Cytokine and chemokine profile were determined in the culture supernatants after 48 h of maturation by CBA. Three individual donors are shown. (TIFF 1597 kb)

Additional file 4: Figure S4. Cytokine-activated NK cells mediate their help for DC maturation via IFN-γ. NK cells were activated for 16 h in the presence of IL-18 (100 ng/ml) and IL-2 (1000 U/ml). Cell-free supernatants were harvested after overnight incubation and added to iDC supplemented with IL-4 and GM-CSF. Blocking antibodies were added where indicated (x-axis). The negative control (− ctrl) represents iDC which have been matured in the presence of IL-2 and IL-18 without NK cell-derived soluble factors. Data are shown as mean of 11 independent experiments. Mann-Whitney U test comparing differences between untreated DC and blocking conditions. ** *P* ≤ 0.01. (TIFF 1481 kb)

## Abbreviations

APC: Allophyocyanin
APC-H7: Allophyocyanin H7
CBA: Cytometric bead array
DC: Dendritic cell
FITC: Fluorescein isothiocyanate
iDC: Immature DC
NK cell: Natural killer cell
PAMP: Pathogen-associated molecular pattern
PE: Phycoerythrin
PerCP: Peridinin chlorophyll protein
PRR: Pattern recognition receptor
TLR: Toll-like receptor
