# Supplementary figures and images for: Pathogen recognition by NK cells amplifies the pro-inflammatory cytokine production of monocyte-derived DC via IFN-γ
Source: BMC Immunol. 2018 Feb 13;19:8. doi: 10.1186/s12865-018-0247-y (PMC5810032; doi:10.1186/s12865-018-0247-y)

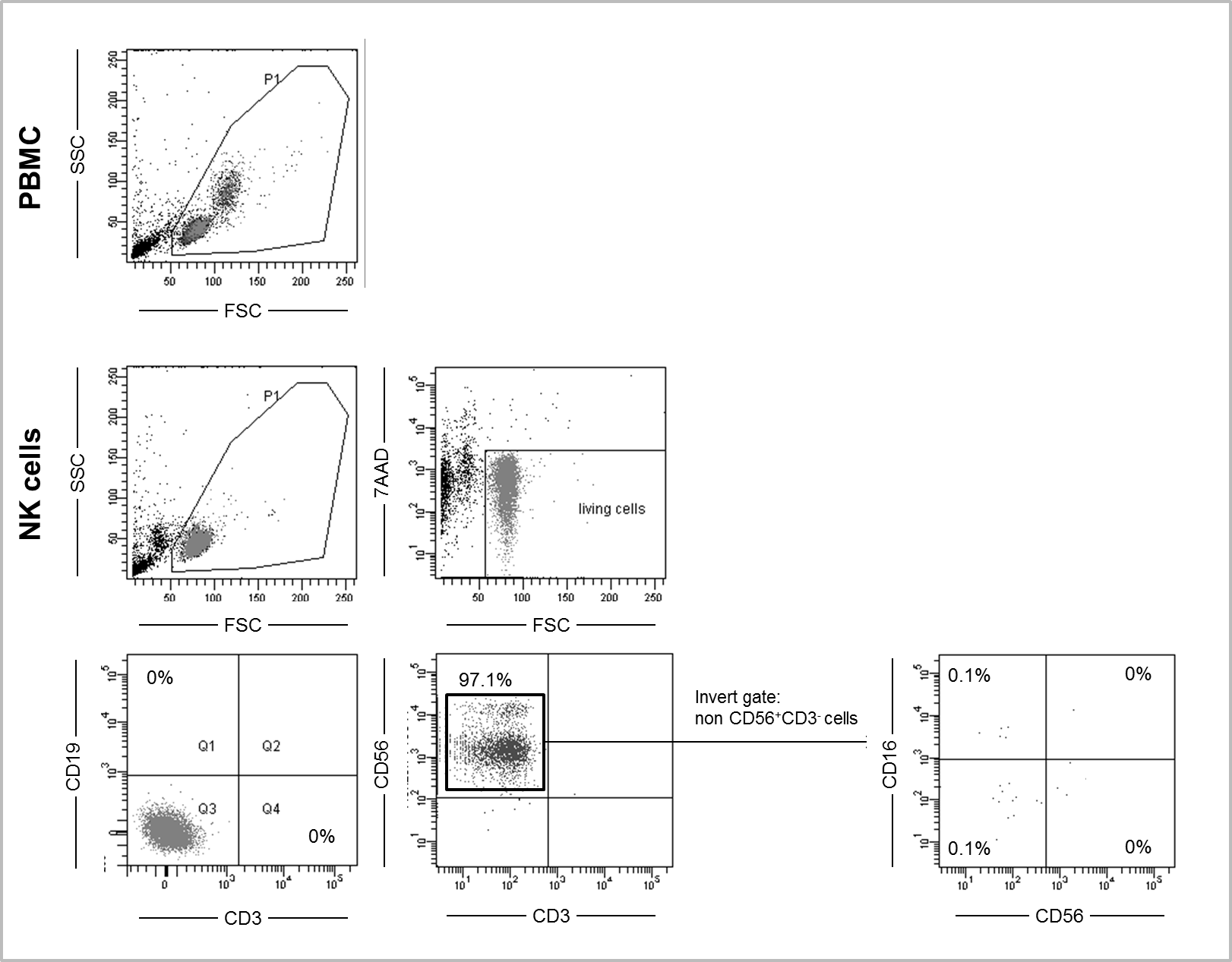

Supplement: Supplementary file 1 — Flow cytometric analysis of NK cell purity. NK cells were gated in the FSC/SSC on the lymphocyte gate and dead cells were excluded by live/dead staining (7-AAD). Percentages of CD19+, CD3+, CD56+CD3−, CD56−CD16+ are indicated in the plots. (TIFF 274 kb) [file 12865_2018_247_MOESM1_ESM.tif]
